# Intraoperative Bronchospasm and Future Asthma in Children: A Retrospective Matched Cohort Study

**DOI:** 10.1111/pan.70029

**Published:** 2025-08-04

**Authors:** Anila B. Elliott, Elizabeth Jewell, Yuan Yuan, Kevin Tremper, Milo Engoren

**Affiliations:** ^1^ C.S. Mott Children's Hospital Ann Arbor Michigan USA; ^2^ Department of Anesthesiology University of Michigan Ann Arbor Michigan USA

**Keywords:** asthma, bronchospasm, general anesthesia, pediatrics

## Abstract

**Background and Objectives:**

Asthma is the most common chronic disease in children. Difficulty in diagnosis can lead to decreased quality of life and increased morbidity and mortality. Children with asthma have increased intraoperative bronchospasm; however, it is unclear whether intraoperative bronchospasm predicts future asthma. We explored intraoperative bronchospasm and subsequent asthma diagnosis.

**Methods:**

We retrospectively analyzed 44,284 children aged 2–18 years who underwent non‐cardiac surgery under general anesthesia between 2014 and 2020. We collected demographic and peri‐operative data, including the occurrence of bronchospasm. We then conducted a subgroup analysis of 35 770 patients that received positive pressure ventilation, using logistic regression to assess the relationship between bronchospasm and airway pressures. The association of bronchospasm and subsequent asthma diagnosis was estimated using generalized estimating equations.

**Results:**

Intraoperative bronchospasm occurred in 128 patients (0.3%) and was associated with increased risk of asthma (OR 2.29, 95% CI 1.10–4.74, *p* = 0.03). Asthma was diagnosed in 1238 patients (2.8%); 8 had intraoperative bronchospasm (8 of 1238, 0.7%). After adjustment for confounders, male sex (OR 1.57, 95% CI 1.39–1.76, *p* < 0.001) and younger age (OR 0.96, 95% CI 0.94–0.97, *p* < 0.001) were also associated with future asthma diagnosis. In the subgroup analysis, Mean PIP (OR 1.50, 95% CI 1.30–1.74, *p* < 0.001) was associated with asthma.

**Conclusions:**

This study shows intraoperative bronchospasm is associated with an increased risk of future asthma in children. Enhanced collaboration between pediatric anesthesiologists and pediatricians, and further research, is essential to improve asthma detection, risk stratification, and overall care for pediatric patients.

## 1 Introduction

Asthma is the most common chronic disease among the pediatric population, with an overall prevalence of 6.5% according to the Centers for Disease Control and Prevention [1], with up to 17% of children in the US afflicted by asthma [2]. It is typically characterized by respiratory symptoms, specifically wheezing, shortness of breath, chest tightness, and cough [3]. In infants and toddlers, symptoms can present slightly differently with tachypnea, chest retractions, nostril flaring, persistent cough, difficulty with feeding, and fatigue. Despite its prevalence, asthma remains challenging to diagnose and there are likely many children who remain undiagnosed [4, 5, 6]. Undiagnosed asthma can lead to increased psychosocial and medical co‐morbidities including chronic obstructive pulmonary disease (COPD), higher rates of absences from school, increased hospitalizations and emergency department visits, and an increased risk of pulmonary complications related to irritants, exercise, or other environmental triggers. These airway irritants include volatile anesthetics and airway manipulation such as an endotracheal intubation, which can lead to bronchospasm during procedures that require anesthesia [7, 8, 9, 10, 11]. Early identification, diagnosis, and treatment of asthma/bronchospasm is important in order to minimize both short‐and long‐term negative impacts on health outcomes [12, 13].

Perioperative bronchospasm in the pediatric population has an incidence between 0.3% and 3.2%, with considerable variation across different centers, with the majority occurring intra‐operatively [13, 14]. A previous study has demonstrated that asthmatic patients have a higher incidence of intraoperative bronchospasm compared to non‐asthmatic patients [15]. However, it has not been shown whether intraoperative bronchospasm is a predictor for future diagnosis of asthma. We aimed to better understand the relationship between intraoperative bronchospasm and postoperative diagnosis of asthma. We hypothesized that intraoperative bronchospasm is associated with an increased incidence of subsequent asthma diagnosis in pediatric patients.

## 2 Methods

We obtained Institutional Review Board approval for this single‐center, retrospective cohort study, which waived patient or guardian consent for the use of deidentified data. Using the institution's data warehouse, we identified pediatric patients ages 2–18 years who underwent surgery with general anesthesia between July 1, 2014, and June 30, 2020. We obtained basic demographic and pre‐operative data including age, gender, height, weight, procedure, and comorbidities, such as recent respiratory infection, obstructive sleep apnea (OSA), asthma, bronchopulmonary dysplasia (BPD), prematurity, eczema, and allergies. We also obtained details pertaining to the anesthetic, including inhalational versus intravenous induction, use of endotracheal tube, medications administered, and intra‐operative complications.

Inclusion criteria comprised the following: (1) Pediatric surgical patients aged 2–18 years who underwent general anesthesia, as younger patients have a known predisposition for bronchospasm and respiratory adverse events in the perioperative period compared to older children [14, 16, 17]. (2) No prior history of asthma, and (3) Received at least one follow‐up visit with a University of Michigan primary care physician (PCP) prior to August 1, 2020. Exclusion criteria included the following: (1) Previous diagnosis of asthma, (2) History of lung disease, including obstructive sleep apnea (OSA), bronchopulmonary dysplasia (BPD), prematurity defined as < 30 weeks gestational age (GA) at birth, as these children have the highest risk of BPD and physiologically tend to have the most respiratory issues related to decreased surfactant [18], congenital diaphragmatic hernias (CDH), congenital pulmonary airway malformation/congenital cystic adenomatous malformation (CPAM/CCAM) or other lesions with significant pulmonary hypoplasia, (3) History of airway defects, (4) Cystic Fibrosis, (5) Airway surgery specific to the larynx and distal to the larynx, (6) Open heart procedures to exclude any airway manipulation or defects affecting the airway, such as vascular rings or prolonged endotracheal intubation, and (7) Allergies, specifically food and seasonal allergies, given the predisposition to reactive airway disease [19]. Our outcome was new postoperative asthma diagnosis.

### 2.1 Case and Control Definitions

The main analysis was performed on intra‐operatively documented “bronchospasm” defined by the following criteria: documentation of bronchospasm in the anesthetic record and/or intraoperative use of albuterol and/or intravenous epinephrine. To confirm the existence of bronchospasm in patients where the etiology was unclear, individual charts were reviewed by a pediatric anesthesiologist who was blinded to the asthma diagnosis, examining multifactorial markers such as peak inspiratory pressures (PIP), oxygen saturation, medication administration, and timing in conjunction with the clinical findings and clinician notes. The first sensitivity analysis included cases with recorded bronchospasm, intraoperative use of an albuterol metered dose inhaler or intravenous epinephrine at dosages ranging from 0.1 to 1 mcg/kg, with or without the administration of intravenous magnesium, and without documentation indicating another etiology, such as hypotension or anaphylaxis. Since bronchospasm was suspected to be under‐reported in the medical record, we created two additional bronchospasm variables (Highest PIP and Mean PIP). Highest PIP was defined as the highest PIP between the time when PIP was first > 5 cmH_2_O after induction and the first PIP that was ≤ 5 cmH_2_0 within 30 min before anesthesia end. Mean PIP was determined over the same time interval encompassing all nonfiltered breaths. To do this, we created a subgroup of patients who received positive pressure ventilation. We then used logistic regression to estimate based on both peak and mean airway pressures patients those who had versus those who did not have bronchospasm by the main analysis. Of note, the default mode of ventilation on the anesthesia machines at our pediatric hospital is pressure control ventilation.

Fragility index was determined by calculating the number of events that would need to be non‐events in order to no longer have a statistically significant *p*‐value [20].

### 2.2 Statistical Analysis

Initial exploratory data analysis was conducted to obtain descriptive statistics. The balance of covariates and confounders between the asthma and no asthma groups was assessed using standardized differences (StDiff).

In addition to the exposure variable presence of bronchospasm, variables selected a priori for consideration in multivariable modeling included the following: patient age, gender, race, anesthesia induction type, presence of an endotracheal tube, anesthesia time, American Society of Anesthesiologists physical status, body mass index (BMI), and gestational age. All variables were assessed for multicollinearity prior to model construction using Pearson correlation coefficients and variance inflation factors.

To identify Highest and Mean PIP values which might be associated with bronchospasm in patients receiving intraoperative positive pressure ventilation, those without bronchospasm were matched based on age, sex, ASA physical status, and emergency status 5:1 to those with bronchospasm. Then, using logistic regression, we used Youden's index to identify a threshold of both Highest and Mean PIP suggestive of bronchospasm.

Multiple imputation using chained equations to impute missing values was used to generate five completed datasets, and results of subsequent analyses were pooled using Rubin's Rules.

To assess the association between bronchospasm and the primary outcome of asthma diagnoses, we used generalized estimating equations models both for the main analysis of all patients and for the subgroup sensitivity analysis of patients receiving positive pressure ventilation. Both analyses adjusted bronchospasm for the confounders. A parsimonious model was decided upon using variables with a *p* < 0.1 from the full model. Odds ratios and 95% confidence intervals were calculated, and goodness‐of‐fit was assessed using areas under the receiver operating characteristics (AUROC) and precision recall curves (AUPRC) [21]. *p*‐values < 0.05 and 95% confidence intervals of the odds ratios that excluded 1 were considered statistically significant. Variables with a StDiff > 20% represented clinical relevance and *p*‐values < 0.05 signify statistical significance. Analysis was performed using statistical analysis system programming (SAS) v. 9.4 (SAS Institute, Cary, NC).

## 3 Results

**FIGURE 1:**
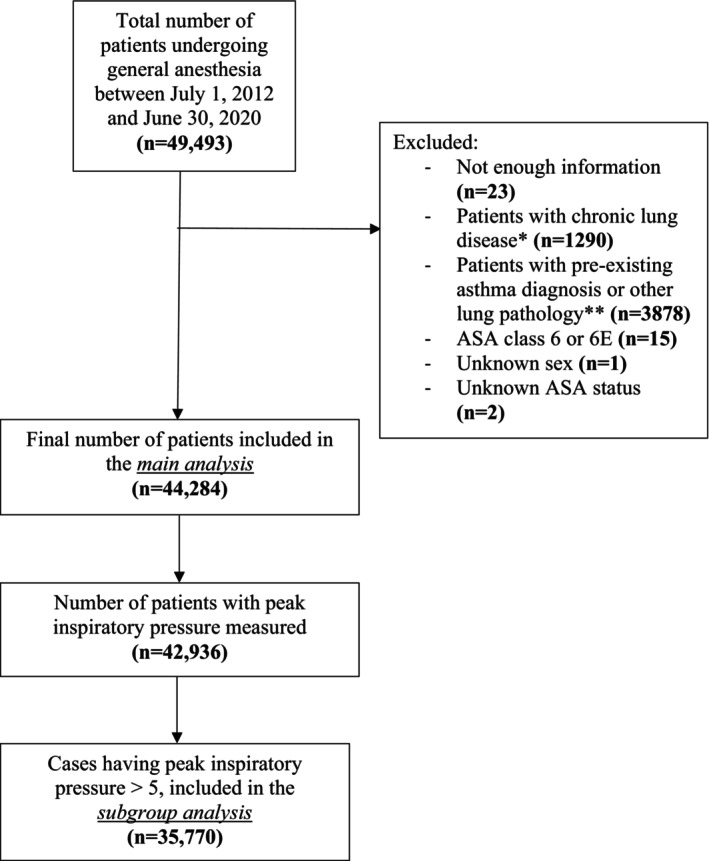
Flowchart of patient inclusion and exclusion criteria in the main and subgroup analysis. *including Bronchopulmonary dysplasia (BPD), prematurity, congenital diaphragmatic hernia. **including obstructive sleep apnea (OSA).

In our main analysis, after excluding 5209 patients, we studied a total of 44 284 patients undergoing non‐cardiac surgery under general anesthesia (Figure 1). Most patients were male (*n* = 22 814, 51.5%), White (*n* = 35 848, 81.0%), and ASA 1 or 2 (*n* = 33 535, 75.7%) [Table 1]. Only 3114 (7%) were emergency cases. Patients' ages ranged from 2 to 18 years with a mean (standard deviation) age of 10 years. (5). About half (*n* = 20 761, 46.9%) of patients had an endotracheal tube. Bronchospasm occurred in 128 patients (0.3%). Asthma was subsequently diagnosed in 1238 patients (2.8%) at a mean follow‐up of 1.55 years. (1.34). Patients with a new asthma diagnosis were more likely to be male (60.8% vs. 51.3%, StDiff = 19.4%, *p* < 0.001), ASA 3 or higher (43.2% vs. 23.7%, StDiff = 57%, *p* < 0.001) and less likely to have an endotracheal tube (37.7% vs. 47.1%, StDiff = 19.2%, *p* < 0.001). Bronchospasm was present in eight patients who subsequently were diagnosed with asthma and in 120 patients who did not receive a diagnosis of asthma (8 of 1238, 0.7% vs. 120 of 43 046, 0.3%, Stdiff = 5.4%, *p* = 0.02).

**TABLE 1:** Descriptive characteristics of the sample: main analysis.

	Total (*N* = 44 284)		Asthma (*n* = 1238)		No asthma (*n* = 43 046)		StDiff	
Variable	Mean	SD	Mean	SD	Mean	SD	%	*p*
Age (years)	10.0	5.3	9.4	5.2	10.0	5.3	11.6	< 0.001
BMI (kg/m^2^)	20.0	5.6	20.3	6.0	20.0	5.6	5.2	0.06
Total Anesthesia time (min)	119.1	109.7	101.2	103.7	119.6	109.8	17.3	< 0.001

	*N*	%	*N*	%	*N*	%		
Sex								
Male	22 814	51.5	753	60.8	22 061	51.3	19.4	< 0.001
Female	21 470	48.5	485	39.2	20 985	48.8		
Race/Ethnicity								
White + Middle Eastern	35 848	81	1007	81.3	34 841	80.9	2.1	0.91
Black + biracial	3745	8.5	100	8.1	3645	8.5		
Asian + American Indian	1615	3.7	48	3.9	1567	3.6		
Unknown race	3076	7.0	83	6.7	2993	7.0		
ASA classification								
1	15 483	35.0	176	14.2	15 307	35.6	57.0	< 0.001
2	18 052	40.8	527	42.6	17 525	40.7		
3	9851	22.3	473	38.2	9378	21.8		
4 & 5	898	2.0	62	5.0	836	1.9		
Emergency	3114	7.0	64	5.2	3050	7.1	8.0	0.01
Induction type								
Intravenous	25 683	58	750	60.6	24 933	57.9	5.4	0.06
Mask	18 601	42	488	39.4	18 113	42.1		
Endotracheal tube present	20 761	46.9	467	37.7	20 294	47.1	19.2	< 0.001
Intra‐operative bronchospasm	128	0.3	8	0.7	120	0.3	5.4	0.02

Abbreviations: ASA, American Society of Anesthesiologists; BMI, body mass index; SD, standard deviation; StDiff, standardized differences.

In the main analysis, we found several factors to be independently associated with an increased likelihood of a subsequent diagnosis of asthma [Table 2]. After adjustment for confounders, we found that intraoperative bronchospasm was associated with a doubling of the risk of asthma (OR 2.29, 95% CI 1.10–4.74, *p* = 0.03). We also found that male sex (OR 1.57, 95% CI 1.39–1.76, *p* < 0.001) and younger age (OR 0.96, 95% CI 0.94–0.97, *p* < 0.001) were associated with future asthma diagnosis, while Black race was not. This model had fair discrimination (area under the receiver characteristic operator curve = 0.69 (0.008)) and fair relevance, being 20 times better than a random classifier (area under the precision recall curve = 0.06 compared to 0.003 for a random classifier).

**TABLE 2:** Parsimonious model of factors associated with future diagnosis of asthma: main analysis.

Parameter	Odds ratio	95% CI	*p*
Age (years)	0.96	0.94–0.97	< 0.001
Body mass index (kg/m^2^)	1.03	1.01–1.04	< 0.001
Total anesthesia time (h)	0.87	0.83–0.91	< 0.001
Male sex (ref female)	1.57	1.39–1.76	< 0.001
ASA classification (ref 1)			
2	2.73	2.29–3.24	< 0.001
3	4.50	3.77–5.38	< 0.001
4 and 5	7.96	5.84–10.85	< 0.001
Induction‐type mask (ref intravenous)	0.89	0.78–1.03	0.11
Endotracheal tube present (ref not present)	0.85	0.74–0.98	0.02
Intra‐operative bronchospasm (ref without bronchospasm)	2.29	1.10–4.74	0.03

Abbreviation: CI, confidence interval.

A subgroup sensitivity analysis was performed using mean and peak inspiratory pressure suggestive of bronchospasm among the 35 770 patients who received intraoperative positive pressure ventilation [Figure 1]. Patient demographics were similar to the main analysis [Table 3]. The Mean PIP was 15 mmHg (5), and the Highest PIP was 23 mmHg (9). Subsequently, 950 patients (2.7%) were diagnosed with asthma. The mean time to diagnosis of asthma was 1.60 years (1.32) compared to 3.55 years (1.70) in those without a diagnosis of asthma. Youden's point was 16.6 cmH_2_0 for Mean PIP and 30 cmH_2_0 for Highest PIP. These criteria identified 9781 patients with Mean PIP ≥ 16.6 cmH_2_O and 4603 had a Highest PIP ≥ 30 cmH_2_O. Patients who subsequently developed asthma were more likely to have Mean PIP ≥ 16.6 cmH_2_O (359 of 950, 37.8% vs. 9422 of 34 820, 27.1%, StDiff = 23.1%, *p* < 0.001), while the proportions of patients with Highest PIP were similar between the two groups (133 of 950, 14% vs. 4470 of 34 820, 12.8%, StDiff = 3.4%, *p* = 0.29).

**TABLE 3:** Descriptive characteristics of the sample: subgroup analysis of patients receiving positive pressure ventilation.

	Total (*N* = 35 770)		Asthma (*n* = 950)		No asthma (*n* = 34 820)		StDiff	
Variable	Mean	SD	Mean	SD	Mean	SD	%	*p*
Age (years)	9.8	5.2	9.2	5.1	9.9	5.2	13.5	< 0.001
BMI (kg/m^2^)	20.0	5.6	20.4	6.2	19.9	5.6	8.2	0.01
Total anesthesia time (min)	128.5	108.1	108.8	100.6	129.0	108.2	19.4	< 0.001

	*N*	%	*N*	%	*N*	%		
Sex								
Male	18 718	52.3	581	61.2	18 137	52.1	18.7	< 0.001
Female	17 052	47.7	369	38.8	16 683	47.9		
Race/Ethnicity								
White + Middle Eastern	28 909	80.8	775	81.6	28 134	80.8	3.7	0.76
Black + biracial	3025	8.5	80	8.4	2945	8.5		
Asian + American Indian	1341	3.8	37	3.9	1304	3.7		
Unknown race	2495	7.0	58	6.1	2437	7.0		
ASA classification								
1	13 823	38.6	157	16.5	13 666	39.3	58.4	< 0.001
2	14 520	40.6	419	44.1	14 101	40.5		
3	6701	18.7	323	34.0	6378	18.3		
4 & 5	726	2.0	51	5.4	675	1.9		
Emergency	2811	7.9	61	6.4	2750	7.9	5.7	0.10
Induction type								
IV	18 988	53.1	521	54.8	18 467	53.0	3.6	0.27
Mask	16 782	46.9	429	45.2	16 353	47.0		
Endotracheal tube present	20 471	57.2	453	47.7	20 018	57.5	19.7	< 0.001
Bronchospasm (mean PIP > 16.5 cmH_2_0)	9781	27.3	359	37.8	9422	27.1	23.1	< 0.001
Bronchospasm (highest PIP > 30 cmH_2_0)	4603	12.9	133	14.0	4470	12.8	3.4	0.29

Abbreviations: ASA, American Society of Anesthesiologists; BMI, body mass index; PIP, peak inspiratory pressure; StDiff, standardized differences.

In this subgroup analysis, several factors were independently associated with an increased likelihood of a future diagnosis of asthma [Table 4]. After adjusting for confounders, we found that Mean PIP was associated with an increased risk of asthma (OR 1.50, 95% CI 1.30–1.74, *p* < 0.001), but not Highest PIP. We also found that male sex (OR 1.60, CI 1.40–1.83, *p* < 0.001) and BMI (OR 1.03, 95% CI 1.01–1.04, *p* < 0.001) were associated with future asthma diagnosis. This model had fair discrimination (area under the receiver characteristic operator curve = 0.71 (0.009)) and fair relevance, being 23 times better than a random classifier (area under the precision recall curve = 0.07 compared to 0.003 for a random classifier).

**TABLE 4:** Parsimonious model of factors associated with future diagnosis of asthma using bronchospasm as determined by the mean peak inspiratory pressure: subgroup analysis.

Parameter	Odds ratio	95% CI	*p*
Age (years)	0.94	0.92–0.95	< 0.001
Body mass index (kg/m^2^)	1.03	1.01–1.04	< 0.001
Total anesthesia time (min)	1.00	1.00–1.00	< 0.001
Male sex (ref female)	1.60	1.40–1.83	< 0.001
ASA classification (ref 1)			
2	2.65	2.20–3.20	< 0.001
3	4.83	3.97–5.87	< 0.001
4 and 5	8.08	5.72–11.41	< 0.001
Induction‐type mask (ref IV)	0.79	0.67–0.93	0.006
Endotracheal tube present (ref not present)	0.74	0.64–0.86	< 0.001
Intra‐operative bronchospasm (by PIP mean) (ref without bronchospasm)	1.50	1.30–1.74	< 0.001

Abbreviation: CI, confidence interval.

The fragility index for the main analysis was 1, and for the subgroup analysis, the fragility index was 75.

## 4 Discussion

We found that intraoperative bronchospasm was associated with the development of future asthma (OR 2.29, CI 1.10–4.74, *p* = 0.03) Our study findings are the first that we know of to evaluate intraoperative bronchospasm being associated with future asthma diagnosis. While other factors that we also found to be associated with an asthma diagnosis, such as male sex and BMI, have been previously described [22, 23] we found no association between future asthma diagnosis and endotracheal tubes [10, 11]. This difference could be related to deeper depth of anesthetic with an endotracheal tube present or to more direct administration of volatile anesthesia contributing to more bronchodilation in our cohort. Notably, we did not find a significant association between asthma and race, potentially due to the geographic and socioeconomic composition of our patient population, which may differ from broader epidemiological studies [24, 25].

While we found that intraoperative bronchospasm is independently associated with subsequent asthma diagnosis, the sensitivity and positive predictive values are low, limiting its clinical utility. However, parents should be informed of intraoperative bronchospasm as it might prompt discussion and questions about symptoms of asthma that may have been missed or previously ignored. Utilizing this information, one could optimize pathways for those with asthma/reactive airway disease to include breathing treatments pre‐operatively, customization of airway devices, and anesthetic agents. It could also lead to improvement in follow‐up care for patients to be seen by their primary care physician, have their pulmonary status optimized, and asthma diagnosed sooner. Future studies would need to be conducted to determine the utility of this approach.

Other limitations include the retrospective nature of our study and reliance on appropriate intra‐operative documentation. Although our team individually reviewed records to assess multiple clinical factors indicative of bronchospasm, some cases may have been missed. Other differential diagnosis considerations that present similarly to bronchospasm include mucous plugging, anaphylaxis, aspiration, and laryngospasm, complicating clinical interpretation and producing an unknown bias in our results. Additionally, we did not fully explore the temporal distribution of the bronchospasm events or the techniques for maintenance of anesthesia, which could provide valuable insights for future considerations and management strategies in this patient population.

Despite pressure control ventilation being the default ventilation mode on our anesthesia machines, variability in practice and retrospective data collection made further investigation into specific ventilation details unavailable. Additionally, as our hospital is both a community hospital and a tertiary care referral center, some patients may have followed up elsewhere, potentially leading to underreporting. Our study also did not account for the potential impact of secondhand smoke exposure or a family history of asthma, which are known risk factors for bronchospasm. This information was not readily available or consistently captured in our electronic medical records but could be explored in future studies to better understand other environmental risk factors. Although we excluded participants with known respiratory diseases or infections, variability in the timing and clinical manifestations of respiratory tract infections could contribute to an increased likelihood of bronchospasm, posing another potential confounder. We also did not have access to information on exercise‐induced wheezing or wheezing associated with upper respiratory tract infections within the last 12 months, which are known to be predictors of asthma development. Furthermore, although our study suggests that intra‐operative bronchospasm is associated with future asthma diagnosis, we are unable to determine if this represents undiagnosed asthma or an increased risk of developing asthma. Either way, the occurrence of intra‐operative bronchospasm should lead to further evaluation, as prompt diagnosis of asthma would facilitate earlier treatment. Our findings highlight the need for additional research to further elucidate risk factors that could help treat and prevent respiratory events related to asthma both daily as well as in the perioperative period.

In our study, we observed 128 cases of bronchospasm in 44 284 anesthetics or 2.9 per 1000 anesthetics in the main analysis and 122 cases of bronchospasm in 35 770 anesthetics or 3.4 per 1000 anesthetics in the subgroup analysis. This incidence is higher compared to larger studies such as Olsson et al., who found a rate of 1.7 per 1000 anesthetics in a database study of over 100 000 anesthetics [14]. Our focus on pediatric patients, who have a higher incidence of bronchospasm, or differing definitions of bronchospasm may account for our higher rate. We also controlled for pre‐existing risk factors such as allergies, eczema, congenital pulmonary pathology, and prematurity. We conducted thorough chart reviews in specific cases where the database data was unclear, looking specifically for notes confirming wheezing, bronchospasm, or other dialogue that would indicate bronchospasm. We also reviewed medication administered, specifically albuterol and other adjunct medication such as magnesium and/or epinephrine, and analyzed air pressures, pressure change, and oxygen saturation during suspected events.

Future studies could impact asthma screening protocols, aid in earlier diagnosis in pediatric populations, and pave the way for future investigations to determine other predictors for asthma diagnosis.

## 5 Conclusion

Asthma remains one of the most common respiratory diseases in the world and continues to be underdiagnosed. Our study indicates that intra‐operative bronchospasm is associated with an increased risk of future asthma diagnosis in pediatric patients. Further research is essential to validate these findings and identify additional risk factors for early asthma detection and management, which could improve risk stratification of patients in the peri‐operative and post‐operative period and improve overall outcomes of pediatric patients.

## Ethics Statement

This study was reviewed and proved by the Institutional Review Board at the University of Michigan Medicine. Informed consent was waived due to the retrospective nature of the study and the use of de‐identified data.

## Conflicts of Interest

The authors declare no conflicts of interest.

## Data Availability

The data that support the findings of this study are available from the corresponding author upon reasonable request.
